# The Impact of Action Intention Versus Action‐Effect Intention on Auditory Prediction Error Signals

**DOI:** 10.1111/ejn.70485

**Published:** 2026-04-09

**Authors:** Andreas Widmann, Betina‐Christiana Korka, Erich Schröger

**Affiliations:** ^1^ Wilhelm Wundt Institute for Psychology Leipzig University Leipzig Germany; ^2^ Zander Labs München Germany

**Keywords:** action effect, auditory, N1, prediction, prediction error, sensory processing

## Abstract

The human brain anticipates the sensory consequences of an action and generates a prediction error (PE) signal when the intended action effect does not occur. This study investigated auditory event‐related potential PE markers based on whether participants intended to perform a specific action or produce a specific action effect. Participants were instructed to press a left or right button to produce low‐ or high‐pitched tones, following a visual pattern. The instructions, actions and tone sequences were identical for all participants. The visual patterns differed in two groups: In the action‐effect intention group, the visual pattern consisted of ‘notes’ (indicating low/high pitch). In the action intention group, the visual pattern consisted of ‘letters’ (indicating left/right button‐press). In both groups, a button‐press occasionally failed to produce the associated tone (incongruent sounds). The key finding was that these incongruent sounds elicited an enhanced auditory N1 component compared to congruent sounds only in the ‘notes’ group. We propose that participants in the ‘notes’ group selected their actions based on the intended action effect, which induced a predictive sensory representation of the expected tone. A violation of this prediction resulted in the early PE, reflected in the auditory N1. Later PE responses, specifically the N2b and P3 components, were observed in both groups. This suggests that action‐effect associations were represented, and their violation was processed at a conceptual level even in the ‘letters’ group. These results support theories postulating that event representations integrate features of stimuli, actions and their associated outcomes.

## Graphical abstract

Sensory action effects are processed differently depending on the type of action selection. Participants selected button press actions producing low/high tones either by action effect intention (notes; left) or action intention (letters; right). Early, sensory N1 prediction error signals in response to rare, unexpected tones were only elicited if actions were selected by action effect intentions. Later, conceptual N2b/P3 prediction error signals were also observed in case of action intentions.

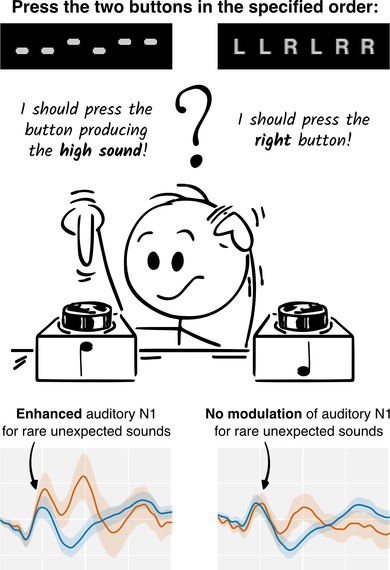

**Abbreviations**
ANOVA: analysis of variance
BF: Bayes factor
CRT: cathode ray tube
dB SPL: decibel sound pressure level
EEG: electroencephalogram
EFA: exploratory factor analysis
EOG: electrooculogram
ERP: event‐related potential
FIR: finite impulse response
ICA: independent component analysis
IR: incongruency response
MMN: mismatch negativity
OSF: Open Science Framework
PE: prediction error
ROI: region of interest
SD: standard deviation
SSA: stimulus‐specific adaptation

## 1 Introduction

When an incoming sound does not match to the internal model, the brain elicits prediction error signals at several levels along the processing hierarchy (Horváth et al. 2008; Wacongne et al. 2011). A plethora of research investigated such signals based on extracted regularities in sound sequences (e.g., base rate and transition probabilities) to gain insight in the brain's predictive processing (for review, see, e.g., Denham and Winkler 2020; Garrido et al. 2013; Meyniel et al. 2016; Näätänen et al. 2001). Interestingly, prediction errors to sounds are also elicited when a sound does not match to the sound an agent intended to produce with a voluntary action (Iwanaga and Nittono 2010; Korka et al. 2019; Nittono 2006; Waszak and Herwig 2007; Widmann and Schröger 2022; for review, see, Korka et al. 2022). This implies that the prediction of a sound cannot be based only on extracted auditory regularities but also on predictions based on the learned coupling between an action and the effect (i.e., the sound) it exerts. It has been proposed that action intention, that is, the goal we want to achieve with our action, can determine prediction in the auditory system (e.g., Korka et al. 2022). Considering that (1) our goals affect the way we represent and process stimuli from our environment (Molinaro and Collins 2023) and (2) that different desired goals can be achieved with the same action (e.g., Hommel 1993), it is to be expected that auditory prediction errors signals are influenced by the action goal. In the present study, we investigated whether the type of intention the participant had when performing an action that generates a tone has an impact on auditory event‐related (ERP) markers of prediction error.

Here, we distinguish between two forms of intention in scenarios with self‐generated tones: the intention to perform an action (which generates a tone) and the intention to produce a tone (which requires an action). We refer to the first type of intention as action intention and the second as action‐effect intention. The hypothesis that the intention type may modulate auditory prediction error signals is fed by cognitive psychology studies revealing an impact of action intention on spatial compatibility effects. For example, the Simon effect (consisting in speeded choice reaction times when the location of a response is spatially congruent with the location of the stimulus to respond to; e.g., Simon and Small 1969) can be inverted when the spatial congruency is on the location of the intended action effect, rather than the actual location of the button to be pressed (Hommel 1993). Although there is an increasing interest in auditory predictive processing in the context of self‐generated sounds, there is (to our knowledge) a lack of research in possible effects of the type of intention on auditory predictive processing.

**FIGURE 1:**
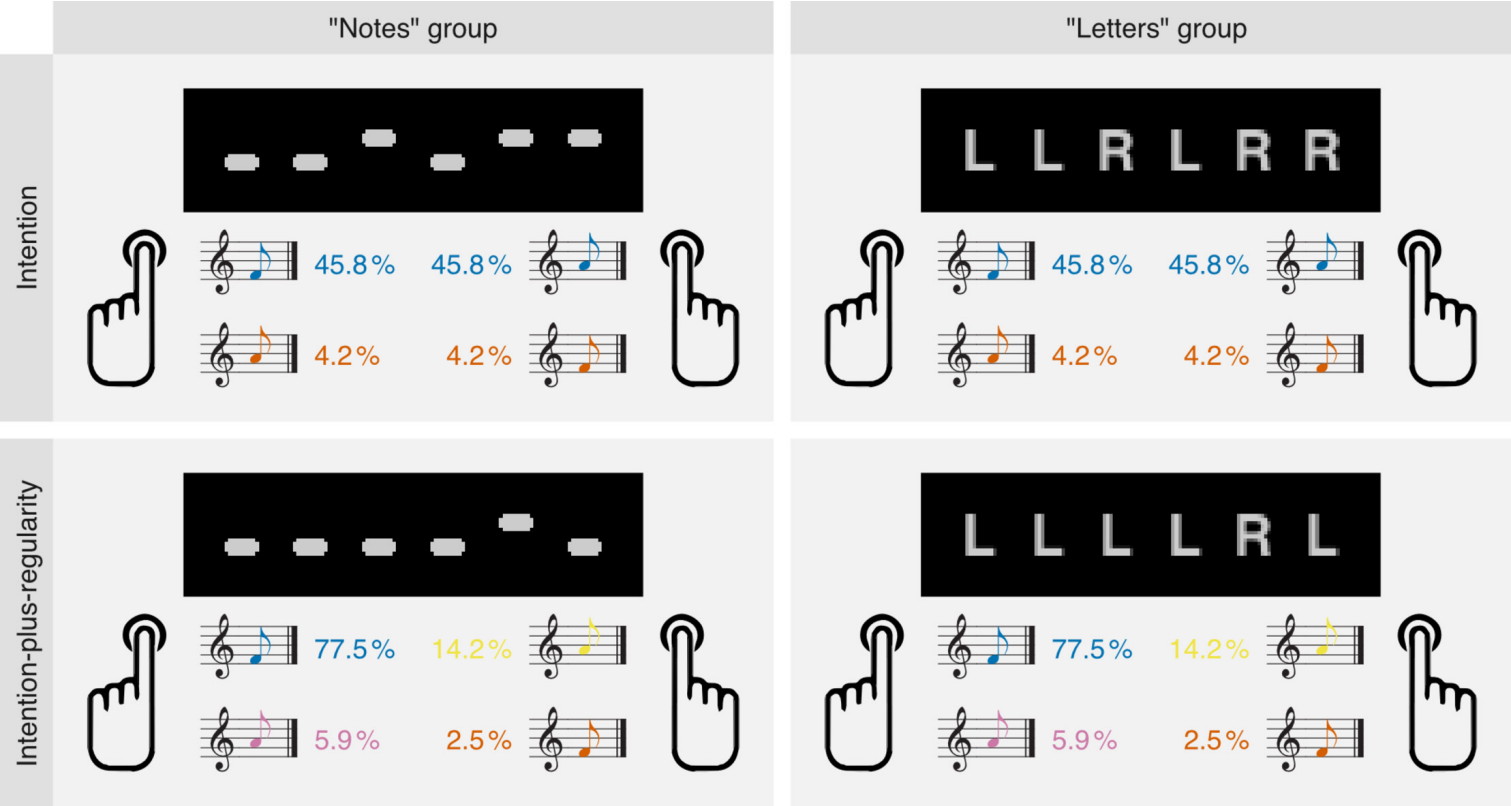
The figure illustrates the experimental design and task. Participants were instructed to ‘play’ a visually presented pattern (comprising six simultaneous note or letter symbols) using button presses. A typical mapping was established: Left button presses usually produced ‘low’ tones (F4, 352 Hz), and right button presses usually produced ‘high’ tones (A4, 440 Hz). Crucially, in 8.4% of trials, an incongruent tone occurred (e.g., left button produced a high tone, or right button produced a low tone). Participants were informed of these rare incongruent tones and instructed to disregard them. Participants were assigned to either the *notes* or *letters* group (between‐subject factor), receiving written instructions that were identical except for the emphasis of visual pattern stimuli (notes vs. letters). Additionally, the relative probability of the to be produced low and high tones was manipulated: In the *intention* condition, participants were instructed to produce an equal number of low and high tones. In the i*ntention‐plus‐regularity* condition, participants were instructed to produce a higher proportion of low tones (83.4%) than high tones. The order of these two conditions was counterbalanced across participants.

Typical ERP effects elicited by a sound not matching to the internal prediction are located at a sensorial level in the N1‐time range (ΔN1/MMN) and at more cognitive, evaluative levels at later time ranges (N2b and P3a). These effects are obtained not only for violations of auditory‐based regularities (for review, see, e.g., Näätänen et al. 2019) but also for violations of action‐effect couplings (Korka et al. 2019, 2021). In previous studies, prediction error responses to violations of expected action effects were implicitly related to action intention by means of instruction (‘produce tones by button presses’, e.g., Korka et al. 2019; Widmann and Schröger 2022; in contrast to ‘press buttons which produce tones’, e.g., Rinne et al. 2001; Waszak and Herwig 2007) but could also be explained by action‐effect couplings. Thus, in order to test for potential effects of the type of intention on these ERP effects, the experimental paradigm not only has to reliably yield those prediction error ERP effects but also must ensure that participants maintain the respective type of intention on each trial. We selected a paradigm where participants produced short sequences of high‐ and low‐pitched tones via left and right button presses according to a pattern of visual stimuli presented on the screen (Figure 1; cf. Widmann et al. 2004). The type of intention (action intention vs. action‐effect intention) was induced by the visual symbols. In the action‐effect intention group, the pattern consisted of simplified note‐like symbols (reflecting the pitch contour of the tones to be produced). In the action intention group, it consisted of the letters L and R (indicating the sequence of left and right button‐presses to be produced). In fact, the action as well as tone sequences produced by the participants were identical in both conditions. Occasionally, a button‐press did not produce the tone (sensory action effect) associated with that button‐press, but it produced an unexpected tone instead. The respective ERP effects were expected to differ between the two conditions. Of special interest are ERP effects in the N1 range, as it reflects sensorial processing in auditory areas: According to some theories, the type of intention may matter for prediction errors located at sensory levels (Schaffner et al. 2023) but not according to others (Mante et al. 2013). Such effects in the N1 time range (termed ‘incongruency response’, IR) were reported previously in a related paradigm by Widmann et al. (2004) using the identical visual displays as in the notes group here, but passively listening to corresponding tone patterns, with the instruction to detect incongruent tones. We hypothesized, to observe an IR in the notes but not in the letters group.

In one condition, low‐ and high‐pitch tones occurred equally often and the prediction errors for unexpected action outcomes were compared between the action‐effect intention (notes) and the action intention groups (letters). In another condition, one tone pitch occurred frequently and the other rarely (tone regularity) so that the effect of regularity of tone pitch in the N1 time range could directly be compared with the effect of action intention (intention‐plus‐regularity condition).

## 2 Methods

### 2.1 Participants

Fifty participants took part in the experiment. One participant had to be excluded due to technical problems during the recording and one participant due to low behavioural performance (less than 50% of the patterns performed correctly). Twenty‐four participants remained in the notes group (mean age 22.7 years, age range 18–28 years, 17 women, 7 men and all right‐handed) and 24 in the letters group (mean age 23.5 years, age range 18–42 years, 14 women, 10 men, 1 left‐handed and 23 right‐handed). All participants reported normal hearing and normal or corrected‐to‐normal vision, and none of them had any history of neurological conditions. The ethics committee of Leipzig University approved the study procedure in accordance with the Declaration of Helsinki. Participants gave written informed consent to participate in the study and received monetary compensation or course credits.

### 2.2 Stimuli and Apparatus

Participants were seated comfortably in a dimly lit, sound‐attenuated and electrically shielded booth. Participants' heads were stabilized with a chin rest. They held two response buttons under the index fingers of their left and right hands. The tones consisted of sine waves with a frequency of 352 Hz (low tone; F4) or 440 Hz (high tone; A4), including the attenuated second (−3 dB) and third harmonics (−6 dB), and had a duration of 300 ms including a 5‐ms rise and 5‐ms fall time (raised cosine window). The tones were presented binaurally via headphones (Sennheiser HD25) at an intensity of 70 dB SPL. A CRT computer screen (G90fB, ViewSonic, resolution 1024 × 768 px, refresh rate 100 Hz) was placed approximately 60 cm in front of the participants so that the visual stimuli appeared slightly below the horizontal line of sight. The visual patterns consisted of six elements, either ‘high’ and ‘low’ rectangles (0.34° × 0.17° visual angle; notes group) placed above or below the horizontal midline or the letters ‘L’ and ‘R’ (0.34° × 0.4° visual angle; letters group) placed along the horizontal midline (see Figure 1). The horizontal spacing between the elements was 0.34°. Together, the note and letter patterns were identical in size and covered a visual angle of 3.7° × 0.4°. Visual stimuli were presented in light grey on a black background. The stimuli were created and presented using Psychtoolbox 3 (Kleiner et al. 2007) and GNU Octave (Version 4) on Ubuntu Linux.

### 2.3 Procedure

The experiment started with a written instruction, the main parts of which were as similar as possible for both groups, except for the graphical illustration of the task (see example visual patterns in Figure 1). The instructions stated that two buttons to be pressed with the index fingers of the left and right hand would produce low (left) and high (right) tones. The task was to reproduce the displayed visual patterns by pressing the buttons in the order indicated by the respective pattern elements: in the notes group, the left button for ‘low’ rectangles (notes group) and the right button for ‘high’ rectangles, and in the letters group, the ‘L’ letters (‘L’ for ‘left’ [‘links’ in German]) or ‘R’ letters (‘R’ for ‘right’ [‘rechts’ in German]). Participants were also told that sometimes the buttons might produce the other tone but that this was irrelevant to the task and should be ignored. Thus, the instruction and the action and tone sequences produced by the participants were virtually identical; only the visual symbols differed between the groups. This was the experimental manipulation to induce a different type of intention between the groups.

Next, participants could look at some example visual patterns and listen to the corresponding tone pattern to learn the tempo at which they would be asked to reproduce the patterns (the tone onset asynchrony in the example patterns was 900 ms). The experiment was divided into an intention and an intention‐plus‐regularity condition, each consisting of four blocks and an additional training block for the first condition. The order of conditions was counterbalanced across participants.

Each block of the intention condition consisted of 54 pattern trials and of the intention‐plus‐regularity condition of 64 pattern trials. The training block consisted of 28 pattern trials. Each pattern trial began with the onset of a visual pattern consisting of six simultaneously presented note or letter symbols. A tone was presented with each button press. In 91.7% of the button presses (11 out of 12), the tone associated with the pressed button was presented (congruent tones; low tone for the left button and high tone for the right button), and in the remaining 8.3% of the button presses (1 out of 12), the other tone was presented (incongruent tones; high tone for the left button and low tone for the right button). All visual patterns consisted of six elements, ‘high’ or ‘low’ rectangles or ‘L’ or ‘R’ letters. The corresponding (identical but ‘translated’) patterns were presented in both groups in a pseudo‐randomized order. In the intention condition, there were never more than three identical subsequent elements. Of the remaining 54 possible patterns, each was presented once in a congruent and once in an incongruent pattern trial per two blocks. In incongruent pattern trials, a single tone was incongruent with the button pressed. The incongruent tones were balanced across positions (the probability of an incongruent tone in the first position was reduced to 3.7% as compared to 9.3% for positions two to six because the first tone of each pattern had to be excluded from the analysis), high and low tones, and tone change and tone repetition. The same number of high and low tones and a total of 108 incongruent and 1188 congruent tones were presented in the intention condition per participant.

In the intention‐plus‐regularity condition, each visual pattern consisted of five identical (frequent) and one different (rare) element. The type of the frequent element (high or low rectangle or L or R) was counterbalanced across participants. Again, in half of the pattern trials, a single tone was incongruent with the button pressed. However, in order to obtain a sufficient number of trials, the probability of frequent incongruent tones (i.e., a rare corresponding visual element) was slightly increased (2.5% instead of 1.4%) and the probability of an incongruent tone at the first position slightly decreased (as in the intention condition). In total, 308 low (or high) and 1228 high (or low) tones, 38 frequent incongruent tones, 90 rare incongruent tones, 218 rare congruent tones and 1190 frequent congruent tones were presented in the intention‐plus‐regularity condition per participant.

If a button was pressed later than 1.4 s after the onset of the visual display or the previous button press, an error message (‘Zu langsam’ [‘Too slow’]) was displayed. If a button was pressed earlier than 0.6 s after the onset of the visual display or the previous button press, an error message (‘Zu schnell’ [‘Too fast’]) was displayed. If a button was pressed that did not correspond to the visual element, an error message (‘Falsche Taste’ [‘Wrong button’]) was displayed. If any of these three error types was detected, the current pattern was terminated, the error was displayed for 1 s and no tone was presented. If all button presses and their timing were correct, the visual display was cleared 0.6 s after the last button press (to avoid contamination of the ERP by a visual change). The next visual pattern was presented after 1 s.

### 2.4 Data Recording

The EEG was recorded with active Ag‐AgCl electrodes from 27 standard positions of the extended 10‐20‐system (Fp1/Fp2, F7/F8, F3/F4, Fz, FC5/FC6, FC1/FC2, C3/C4, Cz, T7/T8, CP5/CP6, CP1/CP2, P7/P8, P3/P4, Pz, O1/O2) and from the left and right mastoids (M1 and M2). All electrodes were referenced to the tip of the nose. The vertical electrooculogram (EOG) was recorded between Fp1 and an infraorbitally placed electrode and the horizontal EOG between the outer canthi of the two eyes. Impedances of all electrodes were kept below 20 kΩ. EEG and EOG were recorded with a time constant of 10 s and sampled with a digitization rate of 500 Hz (BrainAmp, Brain Products, Gilching, Germany). Time was recorded for each button press.

### 2.5 EEG Data Analysis

The first tone of each pattern was excluded from the ERP analysis, as were all congruent tones from patterns containing an incongruent tone. All tones from pattern trials with fast, slow or incorrect responses were also excluded from the analysis.

The EEG data were preprocessed using EEGLAB (Delorme and Makeig 2004). Data were filtered offline with a 48‐Hz low‐pass filter (415‐point Hamming‐windowed sinc FIR filter, transition band width 4 Hz; Widmann et al. 2015) and a 0.1‐Hz high‐pass filter (8251‐point Hamming‐windowed sinc FIR filter, transition band width 0.2 Hz). Data were divided into epochs of 0.7 s time‐locked to tone onset, including a prestimulus baseline of 0.2 s. Channels (except Fp1, Fp2, M1, M2 or EOG channels) were excluded if they had a robust *z* score of the robust standard deviation greater than 3 (Bigdely‐Shamlo et al. 2015; two channels in one participant and a single channel in five participants). Epochs with signals exceeding peak‐to‐peak amplitudes of 750 μV at any electrode were excluded (to remove large nonstereotypical artefacts but to keep stereotypical artefacts as blinks and eye movements to be later removed using ICA). Artefacts were corrected with an independent component analysis (ICA), using the AMICA algorithm (Delorme et al. 2012). For the ICA, the 48‐Hz low‐pass filtered data were filtered with a 1‐Hz high‐pass filter (1651‐point Hamming‐windowed sinc FIR filter, transition band width 1 Hz) and divided into epochs of 0.7 s (removing the same channels and trials as in the previous step) but not baseline‐corrected (Groppe et al. 2009). We then applied the obtained demixing matrix to the 0.1‐ to 48‐Hz filtered data (Klug and Gramann 2021). Artefact ICs were detected with support of the ICLabel plugin (Pion‐Tonachini et al. 2019). All eye‐movement (horizontal and vertical movements of the corneo‐retinal dipoles and presaccadic spike potentials; Plöchl et al. 2012) and blink‐related artefact IC activity, as well as muscle, channel noise or other artefact IC activity causing a significant number of epoch rejections, were subtracted from the data. All artefact ICs were selected manually to minimize the rejection of components showing neural contributions, such as alpha peaks in the spectrum and evoked responses or 1/f‐like power spectra. On average, 7.3 ICs were removed from the data per participant (Mdn = 6; min = 6; max = 13). Bad channels were interpolated using spherical spline interpolation. Data were baseline corrected using the 0.2‐s window before stimulus presentation. Finally, epochs with signals exceeding peak‐to‐peak amplitudes of 200 μV at any electrode were excluded. Individual average ERPs were computed per participant for congruent (mean/median/min/max *N* of included trials per participant 495.1, 508, 339 and 540), incongruent (91.6/94/57/100), rare congruent (112/115/78/122), frequent congruent (375.1/382.5/274/405), rare incongruent (74.6/76/53/80) and frequent incongruent (34.3/35/24/37) tones. Grand average waveforms were computed per group from the individual average ERPs per group and stimulus type.

### 2.6 Statistical Analysis

We conducted temporal exploratory factor analysis (EFA) on the individual average ERP data of all channels and stimulus types using the tutorial code provided by Scharf et al. (2022). EFA was computed using Promax rotation (*κ* = 3) with a covariance relationship matrix (preferable over correlation matrix for ERP analyses as all sampling points are measured on the same scale; for discussion, see Dien et al. 2005; Scharf et al. 2022) and Kaiser weighting (to ensure that each variable has equal influence on the rotation process and therefore prevent that large factors dominate the results of the rotation step; for discussion, see Dien et al. 2005; Scharf et al. 2022). The number of factors to be retained was determined using Horn's parallel test. A total of 16 factors was extracted. We focused our analyses on three factors of interest: N1, N2b and P3a based on the results reported by Widmann et al. (2004).

Mean factor scores were computed within fronto‐lateral (F3, F4, FC5 and FC6; N1) and fronto‐central (Fz, FC1, FC2 and Cz; N2b, P3a) regions of interest (ROI) for each participant and condition. Data were analysed with Bayesian repeated‐measures ANOVAs in JASP (Version 0.19.3; JASP team, 2025; van den Bergh et al. 2023) with the between‐subject factor group (notes vs. letters) and the within‐subject factor congruency (congruent vs. incongruent) with the default prior parameters. Bayes factor estimates for effects were computed using matched models. Post hoc Bayesian paired or independent samples *t* tests were computed in JASP where appropriate (as described in more detail in the results section below). Data were interpreted as moderate evidence in favour of the alternative (or null) hypothesis if BF_10_ was larger than 3 (or lower than 0.33) or strong evidence if BF_10_ was larger than 10 (lower than 0.1). BF_10_ between 0.33 and 3 are considered as anecdotal evidence (following Lee and Wagenmakers 2014).

## 3 Results

### 3.1 Behavioural Data

The ‘notes’ group correctly responded to 89.7% of patterns, compared with 87.8% by the ‘letters’ group. In 1.1% of patterns, a response was given too late (more than 1.4 s after display onset or the preceding button press) by the notes group and in 1.5% by the letters group. In 7.1% of patterns, a response was given too fast (less than 0.6 s after the preceding button press) by the notes group and in 8.9% by the letters group. In 2.2% of patterns, an incorrect response was given by the notes group and in 1.8% by the letters group. The data provided moderate to anecdotal evidence against a difference in performance between groups for all four measures of performance (correct: BF_10_ = 0.335; slow: BF_10_ = 0.338; fast: BF_10_ = 0.368; incorrect: BF_10_ = 0.312).

### 3.2 Intention Condition—N1/IR

**FIGURE 2:**
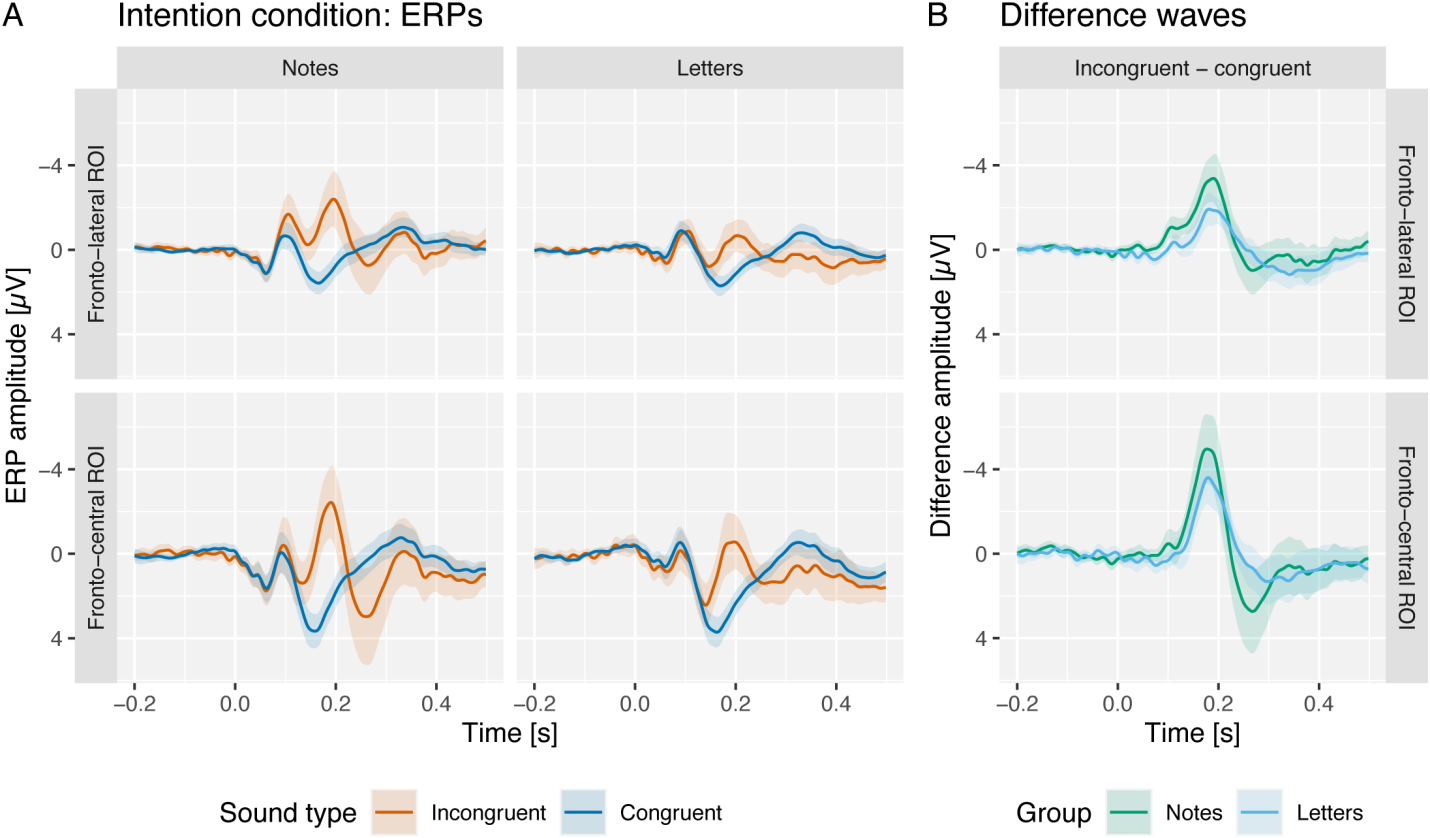
(A) The ERPs elicited in the intention condition in response to congruent (blue) and incongruent (red) tones across the notes and letters groups. Data are presented for two regions of interest (ROIs): fronto‐lateral (F3, F4, FC5 and FC6) and fronto‐central (Fz, FC1, FC2 and Cz). (B) The difference waves (incongruent minus congruent) separately for the notes (green) and letters (blue) groups. The shaded areas indicate the 95% confidence intervals (CIs). A key finding is the elicitation of an incongruency response (IR), characterized by an enhanced N1 component to incongruent tones, which was observed exclusively in the notes group at fronto‐lateral electrode sites.

**FIGURE 3:**
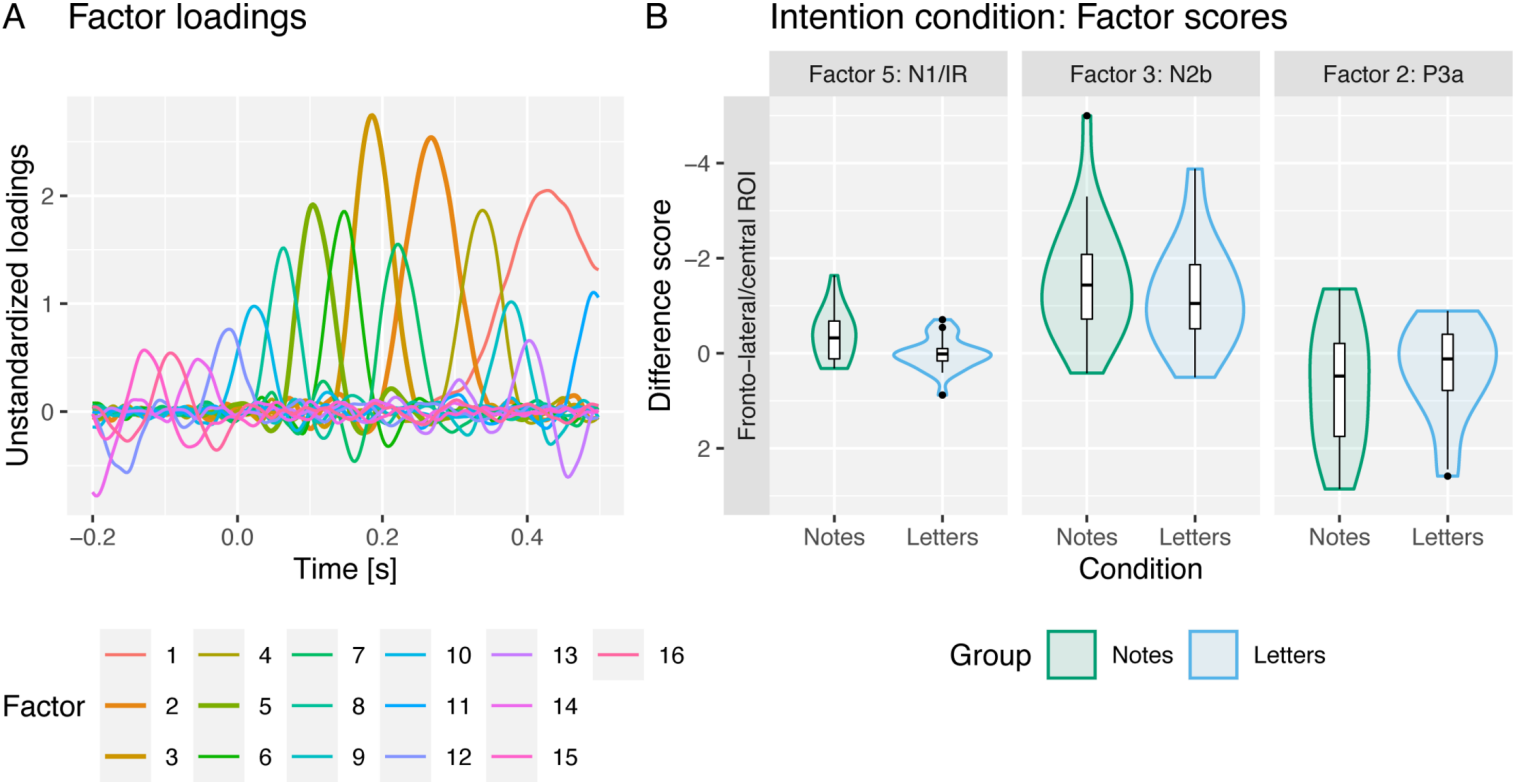
(A) The factor loadings. (B) Violin plots illustrating the differences between the factor scores obtained for incongruent and congruent trials in the intention condition, separately for the notes (green) and letters (blue) groups. These differences are shown for the factors N1/IR (in the fronto‐lateral ROI), N2b and P3a (both in the fronto‐central ROI). An N1/IR was only observed in the notes group. N2b and P3a were observed in both groups.

**FIGURE 4:**
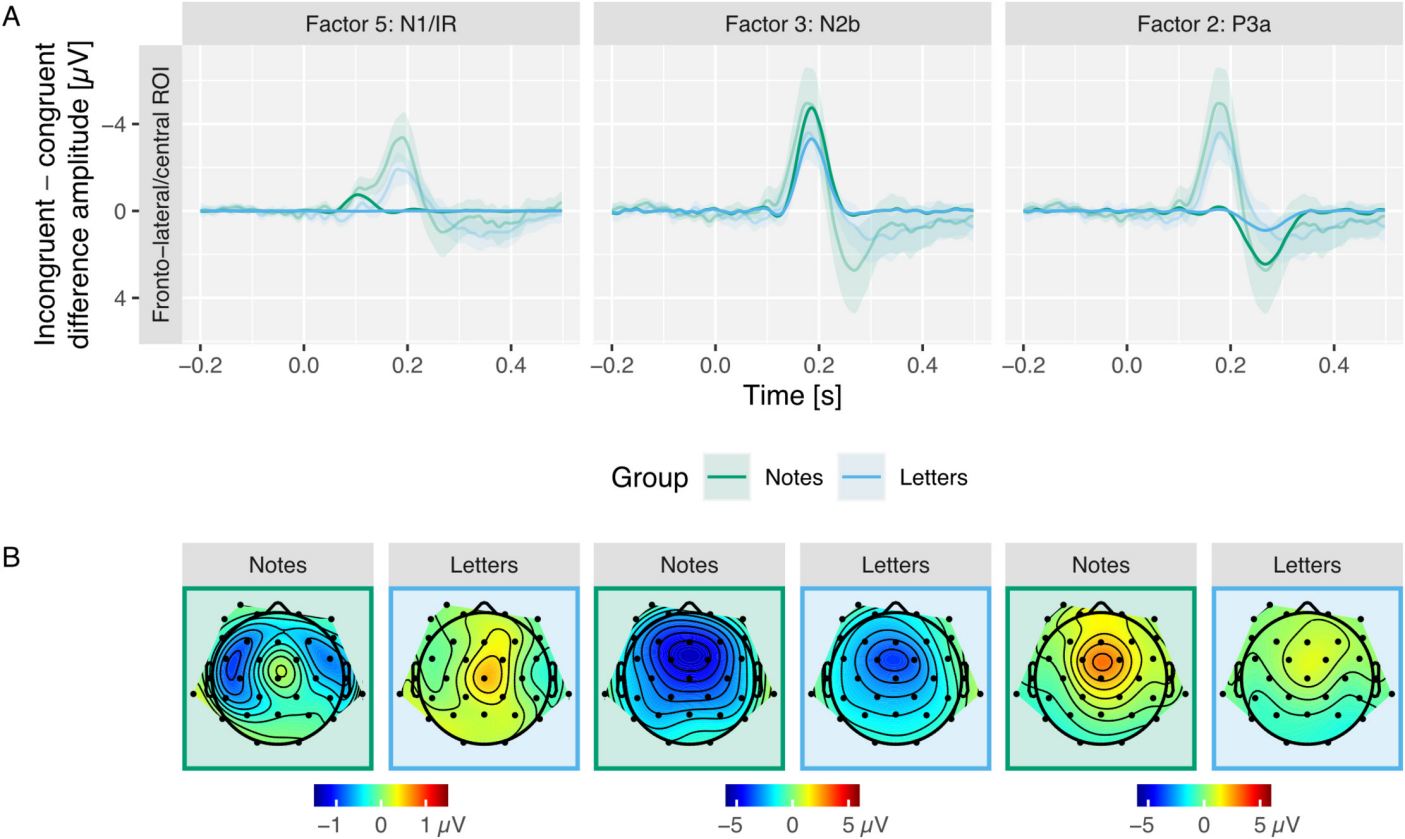
The figure displays the results of the factor analysis comparing incongruent and congruent conditions. (A) The factor time courses, calculated as factor scores multiplied by loadings and the standard deviation, separately for three factors: N1/IR (at 104 ms; factor 5; fronto‐lateral ROI), N2b (at 186 ms; factor 3; fronto‐central ROI) and P3a (at 268 ms; factor 2; fronto‐central ROI). The transparent lines and corresponding confidence intervals (CIs) in (A) depict the grand‐average difference waves (incongruent minus congruent) for the respective groups (notes in green, letters in blue) and ROIs. (B) The topographies of these three factors at their peak latency for both the notes and letters groups.

ERPs in response to incongruent and congruent tones in the intention condition and their difference waves in fronto‐lateral and fronto‐central ROIs are displayed in Figure 2 separately for both groups. Factor loadings and violin plots of factor scores of N1/IR, N2b and P3a components are displayed in Figure 3. Incongruent minus congruent factor time courses and topographies are shown in Figure 4.

The amplitude of the N1 component was enhanced in response to incongruent tones compared to congruent tones in the notes group but not in the letter group. This effect of the violation of a to‐be‐expected sensorial effect of an action (i.e., tone) can be regarded as kind of ΔN1 or MMN, usually obtained when a tone violates a tone regularity. However, in previous research investigating the violation of auditory predictions induced by visual symbolic information (‘high’ vs. ‘low’ visual rectangles), a very similar effect, also revealing a bilateral frontal distribution as the present effect (Figure 4), has been reported (Dercksen et al. 2021; Pieszek et al. 2013, 2014; Stuckenberg et al. 2019, 2021; Widmann et al. 2004). This effect has been termed incongruency response (IR) to acknowledge the fact that no violation of an auditory serial regularity is involved but rather an incongruency between a predicted and an actual tone. The labelling of this effect as ΔN1, IR or MMN is not important in the current context as they all have generators in supratemporal auditory areas. In the Bayesian ANOVA, the data preferred the model including both congruency and group main effects and their interaction (BF_10_ = 32.7). The data provided strong evidence for the congruency by group interaction effect (BF_Incl_ = 11.8). In follow‐up paired samples Bayesian *t* tests, the data provided strong evidence for more negative N1 amplitudes in response to incongruent tones compared to congruent tones in the notes group (BF_10_ = 30.7) and moderate evidence against a difference of N1 amplitudes in the letter group (BF_10_ = 0.216).

In an additional Bayesian independent samples *t* test, we compared the N1 amplitudes in response to congruent tones between groups. The data provided moderate evidence against an N1 amplitude difference between the groups (BF_10_ = 0.297).

### 3.3 Intention Condition—N2b

A N2b component was elicited in both groups. The data preferred the model including the congruency main effect only (BF_10_ = 2.8 × 10^8^). The data provided anecdotal evidence against a group main effect (BF_Incl_ = 0.69) and against a congruency by group interaction effect (BF_Incl_ = 0.628).

As the evidence against a congruency by group interaction was limited, we performed two additional post hoc paired samples *t* tests comparing the N2b component amplitudes in response to congruent and incongruent tones separately within each group to verify that N2b was indeed elicited in both groups. The data provided strong evidence for the elicitation of an N2b component in both groups (notes: BF_10_ = 1647; letters: BF_10_ = 1944).

### 3.4 Intention Condition—P3a

A P3a component was elicited in both groups. The data preferred the model including the congruency main effect only (BF_10_ = 10.9). The data provided moderate evidence against a group main effect (BF_Incl_ = 0.312) and anecdotal evidence against a congruency by group interaction effect (BF_Incl_ = 0.653).

As the evidence against a congruency by group interaction was limited, we performed two additional post hoc paired samples *t* tests comparing the P3a component amplitudes in response to congruent and incongruent tones separately within each group to verify that P3a was indeed elicited in both groups. The data provided anecdotal evidence for the elicitation of a P3a component in the notes group (BF_10_ = 2.79) and anecdotal evidence against the elicitation of a P3a component in the letters group (BF_10_ = 0.81). Therefore, the reported results regarding the P3a component should be interpreted with caution.

### 3.5 Intention‐Plus‐Regularity Condition—Factor 5, N1/IR

As no IR component was elicited in the letter group in the intention condition, we focused our analysis of the intention‐plus‐regularity condition on the notes group only.

The amplitude of the N1 component was enhanced in response to incongruent tones compared to congruent tones (IR) and in response to rare tones compared to frequent tones. The data preferred the model including both congruency and regularity main effects (BF_10_ = 839). The data provided moderate evidence against a congruency by regularity interaction effect (BF_Incl_ = 0.299).

**FIGURE 5:**
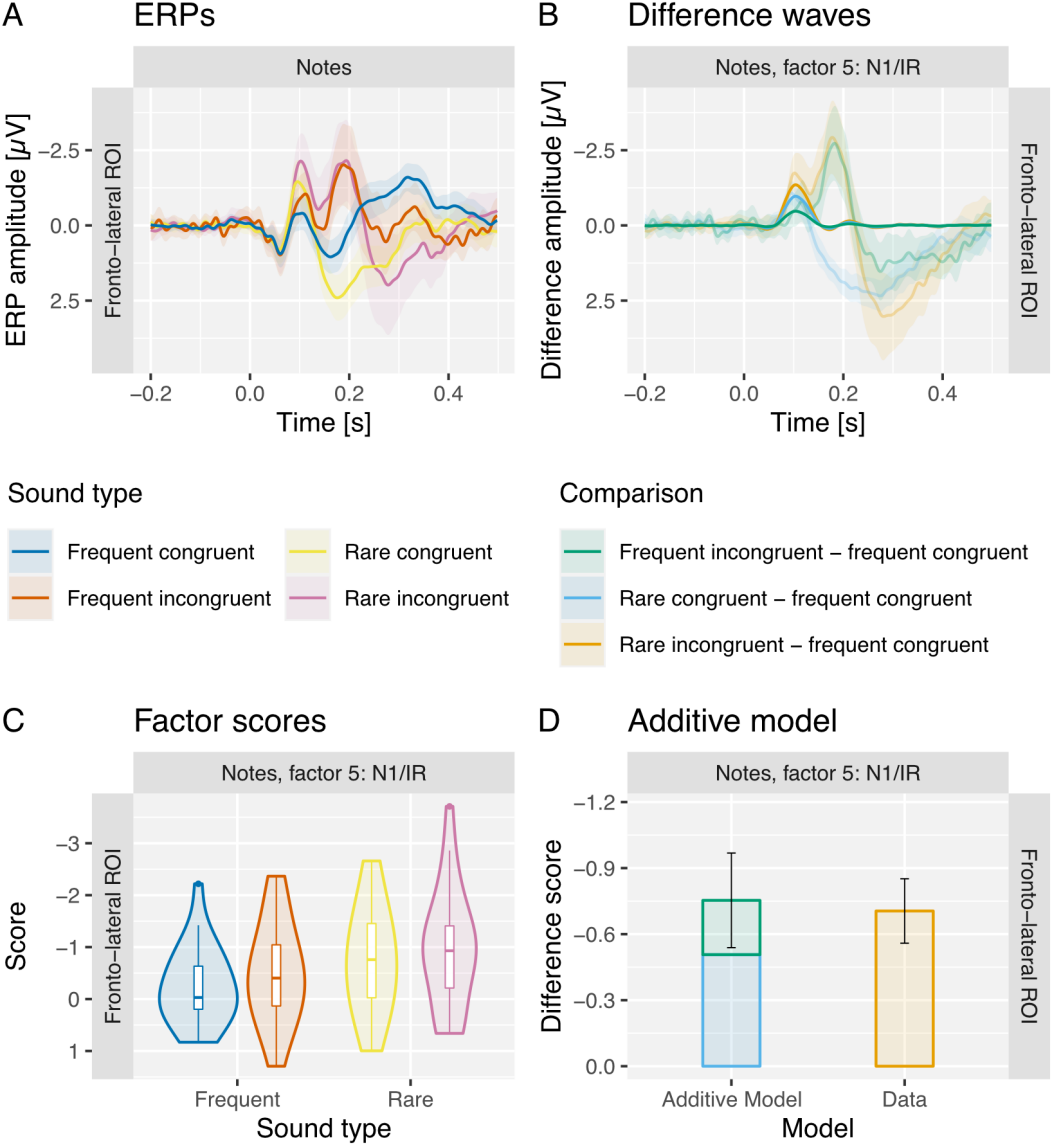
The figure shows the time course and associated scores for factor 5 (N1/IR) in the notes group for the intention‐plus‐regularity condition within the fronto‐lateral region of interest (ROI). (A) The grand‐average ERP time course in response to frequent and rare and congruent and incongruent tones, respectively. (B) The corresponding difference factor time courses (opaque lines) and the grand‐average difference waves (transparent lines). In both (A) and (B), shaded areas represent the confidence intervals (CIs). (C) Violin plots of the associated factor scores. Analysis revealed main effects for both congruency and regularity, but evidence against an interaction effect. (D) The additivity of separate versus concurrent violations of congruency and regularity predictions. Specifically, concurrent violations produced difference amplitudes equivalent to the sum of difference amplitudes from separate violations.

In a Bayesian *t* test, comparing the N1 amplitudes in response to the concurrent violation of both congruency and regularity predictions (rare incongruent minus frequent congruent) against the additive model of separate violations of congruency and regularity predictions [(frequent incongruent minus frequent congruent) plus (rare congruent minus frequent congruent)], the data provided moderate evidence for the null model (BF_10_ = 0.226; cf. Figure 5D). In other words, the data provided evidence that the PE to the concurrent violation is equivalent to the sum of the PE to separate violations of regularity and congruency, which can therefore be assumed to be processed independently.

## 4 Discussion

The present study investigated whether the type of the goal an agent wants to achieve with their action has an impact on event‐related potential (ERP) markers of auditory prediction error. According to research from cognitive psychology, an action can be in the service of different goals, which—in turn—may have an impact on how our brain processes sensorial stimuli associated with these goals (e.g., Herwig and Waszak 2009; Hommel 1993; Molinaro and Collins 2023). In the present study, participants performed short sequences of actions (left/right button presses) resulting in respective short sequences of action effects (high/low pitched tones). In the action intention group (‘letters’), the intention to perform a button press was induced via a graphical representation of the task displaying the letters R (for right button) and L (for left button). In the action‐effect intention group (‘notes’), the intention to produce a tone was induced via a graphical representation of the task displaying ‘high’ (for high pitched tone) and ‘low’ (for low pitched tone) rectangles. Occasionally, the coupling between type of button‐press and type of tone it elicited was violated by presenting the other, unexpected tone.

Previous research showed that the violation of an established coupling between a button press (action) and the sound it generates (action effect) can elicit auditory prediction error signals similar to the ones obtained when a sound violates an established auditory regularity (ΔN1/IR/MMN, N2b, P3a). In an extension of the ‘auditory event representation system’ framework (Winkler and Schröger 2015), Korka et al. (2022) suggested that a prediction for the forthcoming sound cannot be established only on the basis of a learned regularity inherent to the sequency of sounds but also on the basis of learned action‐sound effect couplings. When the generated tone does not meet the prediction, a prediction error is elicited. According to our hypothesis, the prediction and—as a consequence—the prediction error may depend on the type of intention of the acting agent. As auditory predictive processing is hierarchically organized, we tested for prediction error signals at early, auditory sensory and later, cognitive levels. We found evidence for a prediction error at the N1 latency when the actually presented tone was incongruent to the tone the agent intended to generate, whereas there was evidence against such an effect if the agent intended to press the button. The N2b was elicited with both types of intention, whereas the results with respect to the subsequent P3a were inconclusive. In the action‐effect intention group, the prediction errors for violations of tone regularity and action‐effect prediction were additive.

### 4.1 Early, Sensory Prediction Error (N1/IR)

The presence of a ΔN1/IR effect in the action‐effect intention group (notes) suggests that the system noticed the difference between the predicted tone and the actually presented tone at the early sensory level. This implies that the respective representation of the prediction was also sensorial, which is consistent with a proposal from Schaffner et al. (2023), according to which ‘behavioural goals that rely on object perception induce efficient stimulus representations in early sensory structures’. In the action intention group (letters), however, the data provided evidence against the elicitation of a prediction error at the N1 latency. That is, presumably no representation of an auditory sensory prediction was established in this condition. Our partial dissociation between action‐effect intention and action intention prediction errors is furthermore consistent with behavioural research yielding evidence that two independent monitoring processes are involved in evaluating the success of an action (Schaaf et al. 2022): One captures errors in efferent activities (response errors), and one checks for environment‐related irregularities (effect errors).

In order to avoid a potential misunderstanding of the nature of the sensory prediction effects obtained in the present study, it should be stated that our results are neutral in terms of a debate to what extent (motor) forward models are necessary for effective motor control. Forward models are assumed to predict the sensory consequences of actions (Wolpert et al. 1998), to utilize the respective ‘efference’ or ‘corollary discharge’ in order to distinguish between sensory input caused by oneself and by others (Crapse and Sommer 2008) and to explain the emergence of the sense of agency (Haggard 2017). However, according to ideomotor theory‐based ideas of action control (Schreiner et al. 2025), the specification of action goals (i.e., the action‐effect anticipation) is (often) sufficient to activate the respective motor programs and to evaluate the action success by simply comparing the intended action effect with the sensory feedback resulting from the action, without recurrence to internal predictive (motor effect) models. We use the term ‘prediction’ to describe the top‐down processes resulting in a stimulus representation at sensorial (N1) and categorical (N2/P3a) levels, while acknowledging that perception is an inferential process based on predictive generative models (Clark 2013; Friston 2005; Friston and Kiebel 2009). In classical auditory mismatch processing research, the respective (predictive) representation is based on an established auditory regularity extracted from the preceding tone sequence (e.g., transition probability between successive sounds; Schröger et al. 2023), but it can also be based on an established visuo‐auditory coupling (Widmann et al. 2004) and on an action‐effect coupling (present study). So the source of the informational flow activating the representation of the to be expected sound can be in the auditory, visual or action system (cf. Korka et al. 2022). In each case, the resulting auditory representation relates to a forthcoming tone being located in the (immediate) future. In this sense, the underlying processing of information is predictive.

Is the ΔN1/IR effect observed in the action‐effect intention group the result of allocating attention to auditory input? Attention enhances the auditory N1 (Hillyard et al. 1973) and may trigger the endogenous processing negativity at the N1 latency (Näätänen 1982). However, if this were the case, one would expect attentional enhancement for congruent tones in the action‐effect intention group, compared to congruent tones in the action intention group. However, the respective analyses yielded evidence against an N1 amplitude difference between the groups. Thus, differences in the allocation of attention (in the sense of attentional gain or selection or task‐relevance) towards the tones between the groups can presumably be ruled out.

Beyond the question of whether the present effect of instruction can be explained by a difference between groups in the amount of attention allocated to the sounds, there is another facet of attention in the context of predictive coding which is worth considering. In predictive coding theory, attention is conceptualized as weighting of the prediction error by the precision of the sensory information by top‐down gain control (e.g., Feldman and Friston 2010; Brown and Friston 2013; Schröger et al. 2015). Following this concept, one could argue that the prediction error signal reflected in the ΔN1/IR difference in the action‐effect intention group might result from higher weighting compared to the action intention group. Specifically, in the action‐effect intention group the ΔN1/IR prediction error signal demonstrates that a representation of the prediction was established at the respective level and the prediction error sufficiently weighted. Conversely, in the action intention group within the current paradigm, we cannot definitively distinguish whether a prediction was not established or the prediction error was insufficiently weighted. However, we argue that attention, also when understood within the predictive coding framework, does not explain our findings. This argument is based on two facts: First, the actual uncertainty and thus the sensorial precision were perfectly identical across both groups. Second, differences in assumed sensorial precision should also impact the N1 response to congruent sounds; however, the data provided evidence against a difference between groups.

Finally, the present study contributes to resolve a divergency in results of whether the MMN is elicited for self‐generated deviant sounds or not. When participants generated the oddball tone sequence by buttons assigned to the standard and deviant sounds, Rinne et al. (2001) did obtain an MMN, whereas Widmann and Schröger (2022) did not. In fact, the two studies differed with respect to the instruction given to the participants: Whreas Rinne et al. emphasized the button press, Widmann and Schröger (2022) emphasized the production of sounds. In light of the present study, it seems likely that in these two studies the kind of intentional action differed: The study that yielded MMN probably induced action intentions, whereas the study that did not yield MMN induced action‐effect intentions. In the case of action‐effect intentions, a top‐down predictive representation was established at the sensory level, meaning that a self‐generated rare deviant did not violate the prediction. In the case of action intentions, however, no such top‐down representation was established, meaning that rare deviants violated the bottom‐up regularity‐derived prediction, even if they were self‐generated.

### 4.2 Later, Cognitive Prediction Error (N2b, P3a)

N2b is originally reported for targets in the active auditory oddball paradigm (e.g., Ritter et al. 1992). It typically appears around 200–300 ms after sound onset and is linked to conscious detection of irregular task‐relevant sounds. It is also elicited by deviant stimuli triggered by intention‐based actions but not for stimulus‐based actions (le Bars et al. 2019). Similarly, in a study by Iwanaga and Nittono (2010) investigating unexpected action effects in a self‐paced, two‐choice random generation task, the authors reported an increase in N2 (i.e., N2b) when a button‐press infrequently produced a tone violating an established coupling between button‐press and tone. The present study also yielded an N2 for violations of the prediction, thus replicating previous, conceptually similar studies.

Previous studies yielded a P3a for violations of the intended effect of an action (e.g., Darriba et al. 2021; Herwig and Waszak 2009; Knolle et al. 2013; Nittono 2006; Waszak and Herwig 2007; Widmann and Schröger 2022). The present study also yielded statistical evidence in favour of the presence of P3a in the action‐effect intention group indicating the orienting of attention and enhanced evaluation (Escera et al. 1998) of the violation, replicating previous studies. The data were inconclusive as to whether or not a P3a was elicited in the action intention group. The amplitude and effect size of the P3a were relatively small in both groups. It was a fast‐paced task, and participants needed to pay attention to produce the correct sequences. Therefore, less cognitive capacity may have been left for the evaluation process that is reflected in the P3a, and the P3a results should be interpreted with caution.

### 4.3 Additive Processing of Intention‐Plus‐Regularity Violations

In the intention‐plus‐regularity condition, one of the two sounds was presented more frequently, additionally inducing a bottom‐up regularity driven prediction (Garrido et al. 2009; Schröger 1998) besides the top‐down driven action‐effect intention related prediction. The data provided evidence for the additive model, indicating noninteractive, independent processing of both predictions (respectively their violations). This result replicates the important implication that the auditory system may concurrently represent contradictory predictions on the expected sensory input (Pieszek et al. 2013): Even if a rare tone is intentionally generated in the action‐effect intention condition, the regularity‐driven system will still predict a frequent tone.

Other studies also reported that the auditory N1 was not affected by top‐down predictive information. Korka et al. (2019) found no effects of action intention on the N1 (but only on the MMN; interestingly, their data indicated integrative rather than independent regularity vs. intention‐driven effects on the MMN). Similarly, Darriba et al. (2021) reported intention‐related effects only on the later N1b component. Widmann and Schröger (2022) asked participants to produce oddball‐like tone patterns by randomly pressing one button frequently and another button rarely (similar to the present experiment in intention‐plus‐regularity condition, however, with self‐selected rather than visually instructed actions). They found enhanced N1 but no MMN in response to self‐generated oddball deviants (but N1 and MMN in a control condition with randomized button‐tone assignment). A plausible explanation is that different and independent mechanisms underlie the prediction error‐related N1 and MMN responses, with N1 effects contributed to by stimulus‐specific adaption (SSA; Ulanovsky et al. 2003).

It remains an open question whether the additive bottom‐up regularity and top‐down action‐effect intention driven effects in the present study reflect independent N1‐related mechanisms or whether the bottom‐up N1‐related (including SSA) and possibly fast top‐down IR/MMN‐related responses temporally overlap. In any case, this suggests that the brain reveals quite some flexibility in whether it processes prediction errors in the N1 latency range in an interactive or independent manner at an auditory level (N1/IR/MMN). This is best illustrated for the MMN: The action system can turn the MMN system on, that is, an MMN can be elicited in the presence of only a violation of a predicted action‐effect (Korka et al. 2019), or off, that is, when the action system ‘informs’ the tone regularity MMN system that the deviant tone is predicted/intended (Widmann and Schröger 2022). To put the present results in a larger theoretical context, our additivity effects argue in favour of a modularity of the respective auditory regularity and action effect intention driven predictions in the sense of Fodor (1983). However, the underlying processes are presumably not informationally encapsulated at later processing levels, as the effects were not additive in the N2 and P3 latency range. At later, more cognitive processing levels (N2, P3) no additivity of the prediction errors has been reported so far.

### 4.4 Conclusion

In the present study, participants produced tone patterns by sequences of button presses instructed by visual symbols either implying a particular action effect (action‐effect intention or ‘notes’ group) or only relating to a particular action (action intention or ‘letters’ group). We observed an auditory prediction error response to violations of action‐effect associations at the level of sensory processing (auditory N1/IR) in the action‐effect intention group (notes) only, but not the action intention group (letters). A prediction error response related to later, cognitive levels of processing (N2b) was observed in both groups. The elicitation of N2b confirms that the action‐effect associations were indeed represented and processed in both groups. The results are consistent with the ideomotor theory‐based approaches suggesting that actions may be selected based on the anticipation of the associated sensory effect they produce (Prinz 1997; Hommel et al. 2001; Waszak et al. 2012). Importantly, the mere association of action and action effect (which was identical in both groups) is not sufficient, but stronger constraints, such as the goal to produce a particular action effect, are required for action‐effect predictions to be fed back to early sensory levels.

## Author Contributions

**Andreas Widmann:** conceptualization, data curation, formal analysis, investigation, methodology, project administration, software, visualization, writing – original draft, writing – review and editing. **Betina‐Christiana Korka:** conceptualization, writing – review and editing. **Erich Schröger:** conceptualization, funding acquisition, writing – original draft, writing – review and editing.

## Funding

This work was supported by Deutsche Forschungsgemeinschaft (SCHR 375/25‐1).

## Conflicts of Interest

The authors declare no conflicts of interest.

## Data Availability

The aggregated data including the code used for data analysis are provided in the OSF repository (https://osf.io/7vwa8/) in JASP format (including the corresponding html output). The raw data are available from the corresponding author upon request.
